# Artificial Intelligence in Nutrients Science Research: A Review

**DOI:** 10.3390/nu13020322

**Published:** 2021-01-22

**Authors:** Jarosław Sak, Magdalena Suchodolska

**Affiliations:** 1 Chair and Department of Humanities and Social Medicine, Medical University of Lublin, 20-093 Lublin, Poland; 2 BioMolecular Resources Research Infrastructure Poland (BBMRI.pl), Poland; 3 Faculty of Medicine, Medical University of Lublin, 20-059 Lublin, Poland; magda.suchodolska.1998@interia.eu

**Keywords:** artificial intelligence, artificial neural networks, machine learning, nutrients

## Abstract

Artificial intelligence (AI) as a branch of computer science, the purpose of which is to imitate thought processes, learning abilities and knowledge management, finds more and more applications in experimental and clinical medicine. In recent decades, there has been an expansion of AI applications in biomedical sciences. The possibilities of artificial intelligence in the field of medical diagnostics, risk prediction and support of therapeutic techniques are growing rapidly. The aim of the article is to analyze the current use of AI in nutrients science research. The literature review was conducted in PubMed. A total of 399 records published between 1987 and 2020 were obtained, of which, after analyzing the titles and abstracts, 261 were rejected. In the next stages, the remaining records were analyzed using the full-text versions and, finally, 55 papers were selected. These papers were divided into three areas: AI in biomedical nutrients research (20 studies), AI in clinical nutrients research (22 studies) and AI in nutritional epidemiology (13 studies). It was found that the artificial neural network (ANN) methodology was dominant in the group of research on food composition study and production of nutrients. However, machine learning (ML) algorithms were widely used in studies on the influence of nutrients on the functioning of the human body in health and disease and in studies on the gut microbiota. Deep learning (DL) algorithms prevailed in a group of research works on clinical nutrients intake. The development of dietary systems using AI technology may lead to the creation of a global network that will be able to both actively support and monitor the personalized supply of nutrients.

## 1. Introduction

The term “artificial intelligence” was first proposed in 1955 by the American computer scientist John McCarthy (1927–2011) in the proposal of a research project, which was carried out the following year at Dartmouth College in Hanover, New Hampshire [1,2].

Artificial intelligence (AI) as a branch of computer science, the purpose of which is to imitate thought processes, learning abilities and knowledge management, finds more and more applications in experimental and clinical medicine. In recent decades, there has been an expansion of AI applications in medicine and biomedical sciences. The possibilities of artificial intelligence in the field of medical diagnostics, risk prediction and support of therapeutic techniques are growing rapidly. Thanks to the use of AI in ophthalmological [3], radiological [4] and cardiac [5] diagnostics, measurable clinical benefits have been obtained. AI was used in research on new pharmaceuticals [6]. The development of AI also provides new opportunities for research on nutrients and medical sensing technology [7].

### 1.1. Artificial Neural Networks (ANNs)

ANNs as a currently widely used modeling technique in the field of AI were inspired by the structure of natural neurons of the human brain. ANNs are mathematical models designed to process and calculate input signals through rows of processing elements, called artificial neurons, connected to each other by artificial synapses. There are three types of layers forming ANNs. The input layer captures the raw data and passes them to the hidden layer. In this second layer, the learning process takes place. The results of the analysis are collected in the output layer and the output data are created. A neural network may consist of hundreds of single units. An ANN is a parameterized system that has weights as adjustable parameters. Due to the need for estimation of these parameters, ANNs require large training sets. ANNs acquire knowledge by detecting patterns and relationships between data, i.e., through experience, not as a result of programming.

An ANN reveals its particular usefulness in the case of the need for modeling datasets with non-linear dependencies. In solving biomedical problems, raw data can be both literature and experimental data. In the last two decades, ANNs have been used, among others, to create an experimental decision algorithm model open to improvement, aimed at evaluating the results of biochemical tests confronted with both reference values and clinical data [8]. This technique was also used in evaluation of cell culture cross-contamination levels based on mass spectrometric fingerprints of intact mammalian cells [9]. The particular usefulness of ANNs has been proven in pharmaceutical analyses [10]. An interesting application of ANNs is the prediction of the relationship between the Mediterranean dietary pattern, clinical characteristics and cognitive functions [11]. The usefulness of ANNs has been proven in body composition analyses, which have clearly non-linear characteristics [12]. Using ANN modeling, significant benefits can be obtained in clinical dietetics.

It is worth noting that the fuzzy logic methodology (FLM) can be combined with neural networks. The idea of this area of AI is to strive for greater accuracy, dimensionality and simplification of the structure. There is a possibility to create fuzzy neural networks and convert FLM-based models into neural networks.

### 1.2. Machine Learning (ML)

ML is an AI area related to algorithms that improve automatically through experience. ML algorithms have the potential to create mathematical models for decision making. The process of creating these models is based on large sets of training data, without programming. The popularization of the use of ML algorithms took place in the last decade of the 20th century in search engine applications. In the following decades, there were high hopes for significant discoveries in the field of organic synthesis with the use of increasingly advanced ML algorithms [13]. Despite the fact that these hopes have not been fully met, this area of AI has important applications both in biomedical sciences and in clinical medicine. Machine learning—both supervised and unsupervised—can be applied to clinical datasets to develop risk models [14]. It can significantly support the analysis of data obtained from the patient [15].

There are suggestions that ML is the future of computer-assisted diagnostics, biomedical research and personalized medicine [16]. Machine learning techniques are becoming more and more popular in diabetes research: in blood glucose prediction and in the development of the so-called artificial pancreas (a closed-loop system) [17]. The use of ML algorithms in research on the gut microbiota is postulated, especially because of the large datasets collected in these studies [18]. In a recent report, Liu et al. proved that an ML algorithm integrating baseline microbial signatures of the intestinal microbiota can accurately predict the patient’s glycemic response to physical effort [19].

Deep learning (DL) is a subtype of ML. It is an AI domain that has found its applications especially in the techniques of image and voice recognition and foreign language translation. DL also has an important use in medical diagnostics. The significant advantage of DL over supervised ML is expressed in the autonomy of the program in the area of building sets of features used in recognition.

### 1.3. Internet of Things (IoT)

The term IoT was first used by British entrepreneur and startup founder Kevin Ashton in 1999, in the sense of a network of connected objects. This is the concept that objects (devices) can directly or indirectly collect, process or exchange data via a computer network or intelligent electrical installation. The term Internet of Everything (IoE) is used to describe a network of people, processes, data and things connected to the Internet.

In clinical medicine, IoT has a significant application in relation to telemedicine procedures [20,21], which are becoming more and more widely used, especially during the COVID-19 pandemic. Important applications of IoT can also be seen in the provision of detailed information on food products available on the market [22].

## 2. Materials and Methods

The aim of the article is to analyze the current use of AI in nutrients science research and to determine the prospects of its further application in this area.

**Figure 1:**
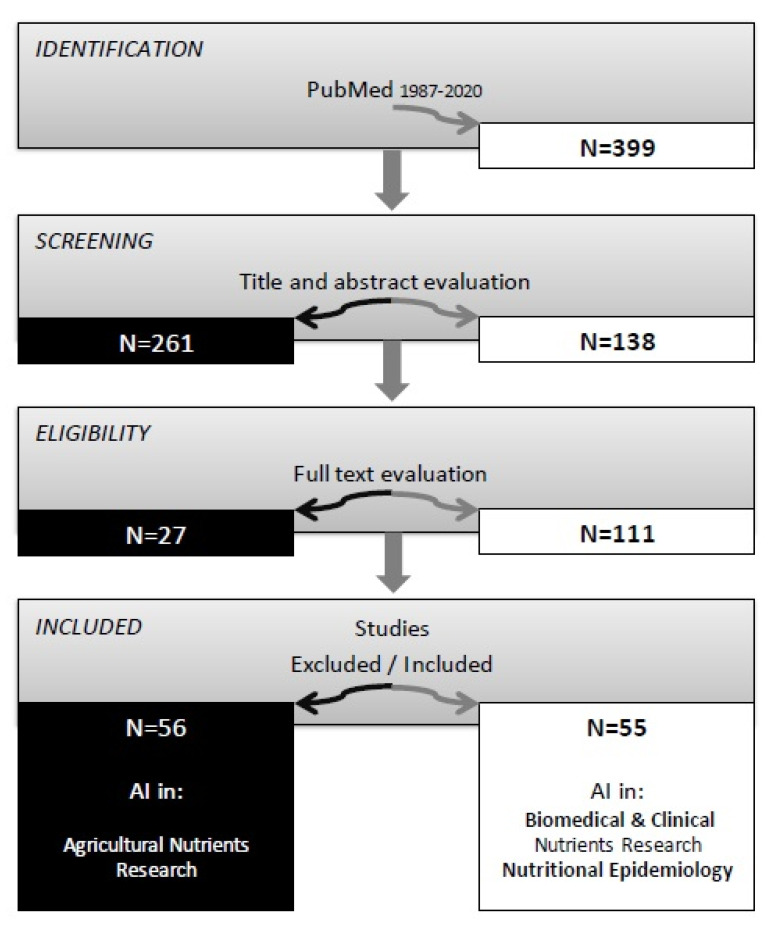
Methodological flowchart of papers reviewed.

The literature review was conducted in PubMed using a combination of searching terms: “artificial intelligence” (All Fields) AND “nutrients” (All Fields). A total of 399 records (published between 1987 and 2020) were obtained, of which, after analyzing the titles and abstracts, 261 were rejected. In the next stage, the remaining records were analyzed using the full-text versions and 111 papers were selected. These papers were afterwards divided into four categories: AI in agricultural nutrients research, AI in biomedical, AI in clinical nutrients research and AI in nutritional epidemiology. In order to limit the analyzed issues to biomedical aspects, agricultural and environmental nutrients research was excluded (Figure 1).

## 3. Results

### 3.1. AI in Biomedical Nutrients Research

In the area of biomedical nutrients research, there were identified studies in which advanced AI methods and systems were applied in relation to the study of the composition of food products, optimization of nutrient production, the effects of nutrients on the functioning of the human body in health and disease and research on the gut microbiota (Table 1).

**Figure 2:**
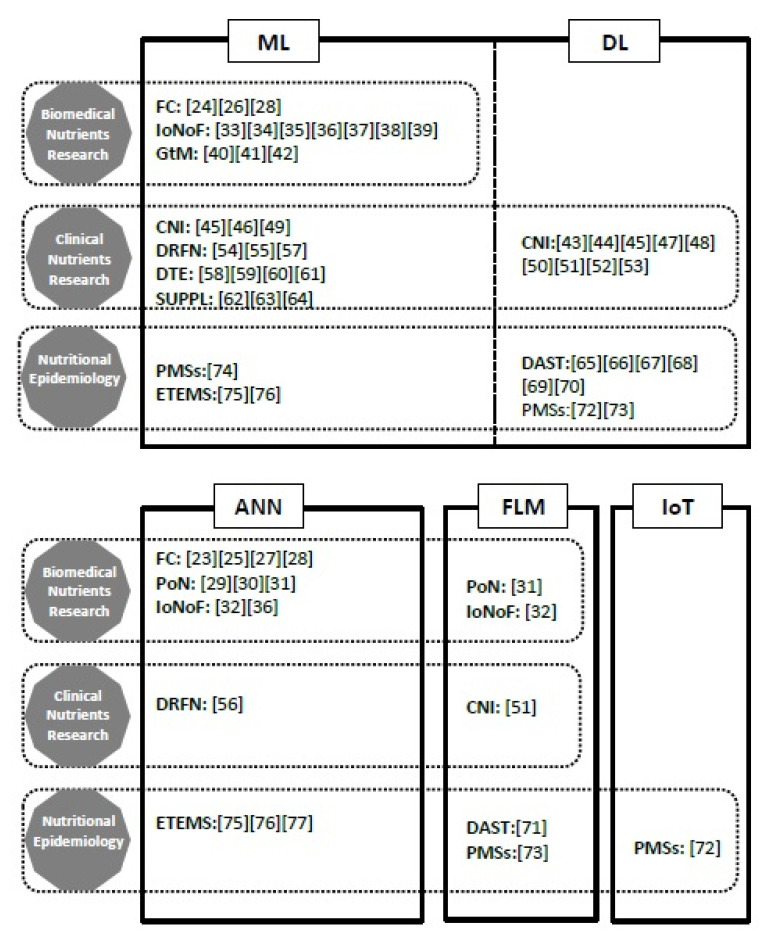
The studies of nutrients science research in relation to artificial intelligence (AI) domains. Note: AI domains: ML = machine learning, DL = deep learning, ANN = artificial neural network, FLM = fuzzy logic methodology, IoT = Internet of Things; biomedical nutrients research: FC = food composition; PoN = production of nutrients; IoNoF= influence of nutrients on phys./path. functions; GtM= gut microbiota; clinical nutrients research: CNI = clinical nutrients intake; DRFN= diseases risks to food and nutrients patterns; DTE = disease and trace elements levels; SUPPL = supplementations; nutritional epidemiology: DAST = dietary assessment; PMSs = physical monitoring systems; ETEMS = environmental trace elements monitoring systems, […] = references.

According to graphical characteristics of the analyzed works (Figure 2), the ANN methodology dominated both in food composition study and the production of nutrients. Among the works on the influence of nutrients on the functioning of the human body in health and disease and studies on the gut microbiota, ML domain algorithms were used almost exclusively. The fuzzy logic methodology was used occasionally.

#### 3.1.1. AI in Food Composition Study

The use of AI techniques in studying the composition of food products and testing their originality dates back to the 1990s. Dettmar et al. used the ANN technique to identify the region of origin of fruit from a set of 16 variables characterizing samples of orange juice [23]. The effectiveness of the applied calculation technique was 92.5%.

Yang et al. used the isobaric tag for a relative and absolute quantification proteomic approach to analyze differentially expressed whey proteins in the human and bovine colostrum and mature milk to understand the different whey proteomes. It may provide useful information for the development of nutrient food for infants and dairy products [24].

Moreira et al. used topological maps of the Kohonen neural network in the assessment of the procedure for sample preparation of cashew nuts [25]. Shen et al. used laser-induced breakdown spectroscopy (LIBS), least squares support vector machines (LS-SVM) and LASSO models for the detection of six nutritive elements in *Panax notoginseng* (traditional Chinese medicine) samples from eight producing areas [26]. Rasouli et al. applied the whole space genetic algorithm-radial basis function network (wsGA-RBFN) method to determine the content of microminerals of Fe^2+^, Zn^2+^, Co^2+^ and Cu^2+^ in various pharmaceutical products and vegetable samples (tomato, lettuce, white and red cabbages) [27]. This group of studies also includes the research of Soltani et al. who used three different quantitative structure bitter taste relationship (QSBR) models (artificial neural network, multiple linear regression and support vector machine) to predict the bitterness of 229 peptides [28].

#### 3.1.2. AI in Research on Production of Nutrients

With regard to research on the optimization of the production of certain nutrients, several studies have been identified in which AI modeling was intentionally applied.

Huang et al. implemented methods of production of a retinol derivative named retinyl laurate by an artificial neural network (ANN) [29]. Zheng et al. studied the optimization of producing 2,6-dimethoxy-ρ-benzoquinone (DMBQ) and methoxy-ρ-benzoquinone (MBQ) as the potential anticancer compounds in fermented wheat germ. They used algorithms of an artificial neural network (ANN) combined with the genetic algorithm (GA) [30]. The ANN model with a Levenberg–Marquardt training algorithm was applied for modeling the complicated non-linear interactions among 16 nutrients in this production process. Kumar et al. used GA-Fuzzy—an evolutionary algorithm comprised of the genetic algorithm (GA) and the fuzzy logic methodology (FLM)—for the optimization of the production of phycobiliproteins (PBPs) from cyanobacteria [31].

#### 3.1.3. AI in Research on the Influence of Nutrients on Physiological and Pathophysiological Functions

The most numerous group of works presenting applications of AI models in biomedical nutrients research is research on vitamins.

Pavani et al. used the neuro-fuzzy model to investigate the influence of alterations in vitamin K (K1, K2 and K3) on modulating the warfarin dose requirement [32]. An AI model was used to predict the warfarin dose, and higher vitamin K1 was observed in the CYP4F2 V433M polymorphism in this study.

The use of AI techniques in research on the influence of vitamin D on the functioning of the human body was described in articles published in 2019. Yu et al. compared the expression profiles of miRNAs, lncRNAs, mRNAs and circRNAs, between 1,25-(OH)_2_D_3_-treated endothelial progenitor cells (EPCs) and control cells. They used bioinformatics analyses to identify differentially expressed RNAs and constructed the competing endogenous RNA (ceRNA) networks with Cytoscape software [33]. Zhang et al. investigated the effect of 1,25-dihydroxy-vitamin D3 (1,25-(OH)_2_D_3_) on primary chondrocytes cultured from patients with an osteoarthritis protein–protein interaction (PPI) by a PPI network [34]. They suggested that their study might provide a theoretical basis for the use of vitamin D in treating osteoarthritis.

Kolhe et al. tried to verify the hypothesis that vitamin C mediates proliferation and differentiation of bone marrow stromal cells through miRNA regulation [35]. They performed bioinformatics analyses to identify novel target genes and signaling pathways. Gene Ontology word clouds were generated using the online Wordle software.

Huang et al. investigated an influence of the active ingredients of licorice (root of *Glycyrrhiza glabra*) for muscle fatigue by RNA-Seq and bioinformatics analysis. They used a machine learning model and a docking tool to predict active ingredients. They identified hispaglabridin B (HB) as a possible inhibitor of FoxO1 which was useful for preventing muscle wasting in chronic kidney disease [36].

Li et al. investigated the effects and mechanism of *Ginkgo biloba* L. on Alzheimer’s disease by using compound-target-disease and compound-group-target-pathway (CGTP) network models [37].

Panwar et al. developed in silico models for predicting vitamin-interacting residues in a protein from its primary structure. They used machine learning techniques such as various classifiers of SVM, RandomForest, BayesNet, NaiveBayes, NaiveBayesMultinomial and ComplementNaiveBayes and position-specific scoring matrix (PSSM) features of protein sequences to identify vitamin-interacting residues in a protein [38]. Yu et al. used a new predictor, the TargetVita web server, and datasets for predicting protein–vitamin binding residues using protein sequences [39].

#### 3.1.4. AI in Research on Gut Microbiota

In recent years, results of research on nutrients and the gut microbiota using AI techniques have been published.

Devika and Raman used genome-scale metabolic models to differentiate between 36 important Bifidobacterial strains [40]. Shima et al. performed analyses concerning the gut microbiota, based on a combination of machine learning and network visualization [41]. Mohammed and Guda used AI in the research on enzymes produced by strains of gut bacteria [42]. They developed ECemble, an approach to identify enzymes and study the human gut metabolic pathways. ECemble uses an ensemble of machine learning methods to predict and identify the enzyme classes. They identified 48 pathways that have at least one bacteria-encoded enzyme and are involved in metabolizing nutrients.

### 3.2. AI in Clinical Nutrients Research

In the past studies in the field of clinical nutrients research, AI techniques have been used in projects aimed at creating tools supporting dietary activities and in supplementation, as well as in the diagnosis and prediction of the risk of chronic diseases (Table 2).

According to the graphical characteristics of the analyzed works (Figure 2), the DL methodology dominated in the group of studies on clinical nutrients intake. A marginal use of the fuzzy logic methodology was noted—it appeared only in one study.

#### 3.2.1. AI in Clinical Nutrients Intake

Among the identified studies on the application of AI in clinical practice, there is a need to distinguish those that aimed to develop systems that monitor, support and modulate the nutrition of chronically ill people. Lu et al. presented a novel system based on AI to accurately estimate nutrient intake, by simply processing RGB depth image pairs captured before and after meal consumption [43]. Oka et al. compared AI-supported nutrition therapy with a mobile application (*n* = 50) versus human nutrition therapy (*n* = 50) in a randomized controlled trial [44]. An interesting technological solution in the AI area was used by Vasiloglou et al. in relation to the clinical problem of controlling carbohydrate intake in patients with type 1 diabetes. These authors used GoCARB as a computer vision-based smartphone system in determining plated meals’ carbohydrate content. In this study, the estimation of carbohydrate content in 54 plated meals made by GoCARB was compared to the estimation made by six experienced dietitians. It was found that GoCARB estimated the carbohydrate content with the same accuracy as professional nutritionists (*p* = 0.93) [45].

Chin et al. tested the Automated Self-Administered 24-Hour Dietary Assessment Tool (ASA24) on the example of lactose with regard to the Nutrition Data System for Research (NDSR) [46]. ASA24, also known as food diaries, is a web-based tool that enables multiple, automatically coded, self-administered 24-h diet recalls. NDSR is a dietary analysis software application widely used for the collection and coding of 24-h dietary recalls and the analysis of menus. Nine machine learning models have been developed based on the nutrients common to ASA24 and the NCC database. The results obtained in this study suggest that computational methods can successfully estimate an NCC-exclusive nutrient for foods reported in ASA24.

In order to monitor eating behaviors, a rapid automatic bite detection algorithm (RABID) that extracts and processes skeletal features from videos was constructed. Konstantinidis et al. used it to analyze the eating behaviors of *n* = 59 patients (three types of dishes, 45 meals), the results of which showed an agreement between algorithmic and human annotations (Cohen’s kappa κ = 0.894; F1-score: 0.948) [47].

Chi et al. proposed a knowledge-based system (KBS) for patients with chronic kidney disease using the Web Ontology Language (OWL) and the Semantic Web Rule Language (SWRL) [48]. In order to evaluate the designed system in recommending appropriate food serving amounts from different food groups, information was collected from *n* = 84 patients. It was found that the OWL-based KBS can achieve accurate problem solving and reasoning questions while maintaining the ability to share and extend the knowledge base.

AI techniques can also be useful in diagnosing mild dehydration. Posada-Quintero et al., using machine learning, investigated the possibility of detecting mild dehydration with autonomic responses to cognitive stress (*n* = 17) [49]. Taking into account the autonomic control indicators based on electrodermal activity (EDA) and pulse rate variability (PRV) in the Stroop test, they obtained 91.2% overall accuracy of mild dehydration detection.

In the area of AI applications in the improvement of dietary solutions, two articles describing prototype solutions should be mentioned. Khan and Hoffmann proposed a menu construction using an incremental knowledge acquisition system (MIKAS) [50]. This system asks the expert to provide an explanation for each of their actions, in order to include the explanation in its knowledge base, so MIKAS could in the future automatically perform them.

Fuzzy arithmetic has been used to create “Nutri-Educ”—software for proper balancing of meals, according to the energy needs of the patient. Heuristic search algorithms are used to find a set of actions, acceptable from a nutritional point of view, that will transform the initial meal into a well-balanced one [51].

Baek et al. applied the hybrid clustering-based food recommendation method that uses chronic disease-based clustering and a nutrition knowledge base [52]. Food products are grouped using the k-means algorithm and food and nutrient data system. Based on the created clusters and data on food preferences, a knowledge base on diet and nutrition is generated.

Mezgec and Koroušić Seljak introduced a new “NutriNet” tool for food image recognition based on a deep convolutional neural network architecture [53]. It was tested on a collection of 225,953 images (512 × 512 pixels) of 520 different foods and beverages. This tool with an implemented training component is used in practice as a part of a mobile app for the dietary assessment of Parkinson’s disease patients.

#### 3.2.2. AI in Evaluating Diseases Risks in Relations to Food and Nutrients Patterns

AI techniques also appear to be useful in estimating the risk of health problems based on the analysis of dietary or supplementation patterns. Panaretos et al. used the k-nearest neighbors algorithm and random forests decision tree to assess the 10-year cardiometabolic risk in relation to nutrient and food patterns, *n* = 3042 (2001–2002) [54]. The authors of the study, using factor analysis, identified factors from foods and nutrients, respectively, explaining 54 and 65% of the total variation in intake. ML techniques were found to be superior compared with linear regression in health score classification.

Berry et al. in *n* = 1002 twins and unrelated healthy adults groups (PREDICT 1 study) assessed the inter-individual variability of postprandial metabolic responses (triglyceride, glucose, insulin) as potential risk factors for cardiometabolic diseases [55]. On the basis of conducted cohort studies, they developed a machine learning model that predicted both glycemic (r = 0.77) and triglyceride (r = 0.47) responses to food intake.

Naushad et al. developed a breast cancer prediction model based on an artificial neural network (ANN) to investigate how micronutrients (foliate, B12) modulate susceptibility to breast cancer [56]. The developed ANN model explained 94.2% variability in breast cancer prediction.

This group of studies also includes the article by Shiao et al., who examined *n* = 106 participants in multi-ethnic colorectal cancer families in terms of prognostic factors of healthy eating (HEI index) [57]. Machine learning validation procedures were applied, including the ensemble method, generalized regression prediction, elastic net and leave-one-out cross-validation methods.

#### 3.2.3. AI in Studying the Relationships between Disease and Trace Elements Levels

In a review of AI application reports, there were identified articles examining the levels of selected trace elements in biological samples collected from patients with type 2 diabetes. Tan et al. examined the usefulness of machine learning (Adaboost) in combination with trace element analysis of hair samples in diagnosing CVD in clinical practice (*n* = 124) [58]. The same authors examined the levels of several elements, including trace elements: lithium, zinc, chromium, copper, iron, manganese, nickel and vanadium, in whole blood of type 2 diabetes patients (*n* = 53), comparing them with analogous data obtained from healthy people (*n* = 105) [59]. In order to construct the model, they used Fisher linear discriminate analysis (FLDA), a support vector machine (SVM) and a decision tree (DT) for data analysis. In 2014, the results of the relationships between several element levels in hair/urine and diabetes mellitus (*n* = 211) were published using ensemble and single support vector machine (SVM) algorithms as the classification tools [60].

In addition to the use of AI techniques in the study of the relationship between the risk of diabetes and trace elements, the study of relationships between schizophrenia risk and serum levels of macro and trace elements should also be noted. Lin et al. for this purpose used samples taken from 114 schizophrenia patients and 114 healthy controls and supervised learning methods [61]. The levels of 39 macro and trace elements were examined and the best prediction accuracies were achieved by support vector machines.

#### 3.2.4. AI in Studying on Supplementations

Li et al., in a recent report, described the performed bioinformatics analysis and computation assays using a network pharmacology method to evaluate the properties of vitamin A as an anti-SARS-CoV-2 regimen [62]. A similar research goal was achieved by the team of Chen et al., who, using network analysis, tested the potential of a novel combination of vitamin C, curcumin and glycyrrhizic acid (VCG Plus) against CoV infection [63]. Further, using network analysis, Fan et al. attempted to identify a molecular mechanism delaying the onset of psychotic symptoms in Alzheimer’s disease in association with the use of vitamin D [64].

### 3.3. AI in Nutritional Epidemiology

In the area of nutritional epidemiology research, there were identified studies in which advanced AI methods and systems were applied in relation to the dietary assessment, physical monitoring systems and environmental trace elements monitoring systems (Table 3).

In this research area, the algorithms of ML and DL were used predominantly (Figure 2). The methodology of ANN was used in environmental trace elements monitoring systems. The application of the IoT methodology was noted in the physical monitoring systems topic.

#### 3.3.1. AI in Dietary Assessment

Mobile applications based on systems using AI are of significant importance in the field of nutritional prophylaxis (Table 3). In 2008, Sun et al. proposed an electronic photographic approach and associated image processing algorithms to estimate food portion size [65]. Lu et al., in a recent publication, offered goFOOD^TM^ as a dietary assessment system based on AI. It can estimate the calorie and macronutrient content of a meal, on the sole basis of food images captured by a smartphone [66].

Yang et al. proposed a new methodological approach in the field of nutritional epidemiology, Ontology for Nutritional Epidemiology (ONE) [67]. It is a resource to automate data integration, browsing and searching. ONE can be used to assess reporting completeness in nutritional epidemiology.

Lo et al. created an objective dietary assessment system based on a distinct neural network [68]. They used a depth image, the whole 3D point cloud map and iterative closest point (ICP) algorithms to improve the dietary behavior management.

Fang et al. estimated food energy based on images and the generative adversarial network (GAN) architecture (*n* = 45) [69].

Ji et al. assessed the relative validity of an image-based dietary assessment app—Keenoa—and a 3-day food diary in a sample of healthy Canadian adults (*n* = 102) [70]. The authors in this randomized controlled trial showed that Keenoa had better validity at the group level than the individual level and it can be used when focusing on the dietary intake of the general population.

Hsu et al. used the fuzzy decision model to develop a web-based support system that searches food composition databases and calculates dietary intake [71]. This research project was carried out due to the lack of integrated databases for Chinese menus and the need for a decision-making tool for dietitians in Taiwan.

#### 3.3.2. AI in Physical Monitoring Systems

AI techniques have found their application not only in monitoring the quality and quantity of nutrients, but also in terms of the level of their expenditure. In the face of the obesity epidemic, these AI applications are very important. Monogaran et al. described the use of a monitoring system as an effective diagnosis tool of physical activities by a wearable smart-log patch with Internet of Things (IoT) sensors [72]. The data were analyzed using edge computing on a Bayesian deep learning network (EC-BDLN). Tragomalu et al. analyzed e-health applications for the management of cardiometabolic risk factors in children and adolescents [73]. Ramyaa et al. tried to phenotype women based on dietary macronutrients and physical activity using machine learning, support vector machine (SVM), neural network and k-nearest neighbors (kNN) algorithms [74].

#### 3.3.3. AI in Environmental Trace Elements Monitoring Systems

Novic and Groselj used an ANN to create a methodology for food specifications associated with the origin of food. The methodology was tested on honey samples collected by the TRACE UE project [75]. The data were collected from various regions of Europe and analyzed for the content of trace elements.

Research on the content of trace elements and rare-earth elements in honey was also carried out by Drivelos et al. [76] They used probabilistic neural network (PNN) analysis and constructed a partial least squares (PLS) model for classifying of honey samples according to their geographical origin and organic characterization.

Tunakova et al. used an ANN to create a neural network model describing the retentions of trace elements in the human body. They calculated the microelement levels in the body, knowing the trace element levels in drinking water and urine [77].

## 4. Discussion

One of the main problems in analyzing publications on the use of AI in nutrient research is the range of research areas to be considered. This type of research creates a very diverse spectrum of problems. They are not limited to the field of biomedical sciences, but also apply to plant and animal breeding, including the breeding of microorganisms. The limitations which were found in the methodology of the review were dictated by the intention to maintain transparency. Therefore, studies that directly or indirectly relate to human health were included, excluding research on nutrients in agricultural and veterinary sciences. The review of the publications revealed three application areas of AI technology: biomedical nutrients research, clinical nutrients research and nutritional epidemiology.

During the analysis of the reviewed publications presenting the results of research on nutrients with the use of AI technology, it can be noticed a little later that it gained wider application in human health research than analogous applications in experimental research on food. This may have resulted from both some ethical concerns and psychological resistance, as well as from the imperfections of earlier AI algorithms, which seemed not yet ready to solve problems concerning the human body. A significant increase in the number of publications on the use of AI in nutrients research has been recorded in the last decade (2011–2020). Perhaps the title question from the article by Gedrich et al., “How optimal are computer-calculated optimal diets?” [78], asked at the end of the last century was significantly ahead of the medical professions’ mentality.

The use of AI in biomedical nutrients research reflects the need for efficient analysis of large datasets that could not be analyzed using traditional statistical methods. This applies in particular to the study of the relationship between nutrients and the functioning of the human body and in the study of the gut microbiota [40,41,42]. The increasing use of AI algorithms in this area is an expression of scientific progress and is becoming not only a privilege, but even a necessity in the pursuit of obtaining valuable results. The possible decoding of the gut microbiota functioning mechanisms can bring significant benefits in the form of possibilities to develop modern and very effective probiotics.

The application of AI algorithms in clinical nutrients research is expressed both by systems supporting dietary activities, diseases risks in relation to food and nutrients patterns and supplementation research. An important issue in this research area is the assessment of the reliability and credibility of the test results obtained using AI techniques. Another essential issue is the modification of the dietician–patient relationship in the case of replacing, in whole or in part, the work of a medical professional by AI systems [43,44,45,46,47,48,49,50,51,52,53]. The problem of trust in AI-based systems, especially in the elderly, remains open. In the social dimension, however, with the implementation of modern technologies in everyday activities, an increase in trust in both robotic systems and AI systems in medicine is observed. Especially on the basis of the articles included in the review, it is possible to state potentially good-quality effects of using dietary AI systems. Comparing them with the assessment of professional nutritionists, it is worth noting that in both cases, there were similar difficulties with regard to estimating the caloric value of some food products (e.g., GoCARB) [45]. The use of AI systems in dietary assessments enables personalized nutrition, which in some diseases is a priority.

The development of AI systems in dietetics may lead, in the near future, to a partial replacement of medical personnel and reducing the need for personal contact with a nutritionist. In the face of contemporary epidemiological threats, this seems to be of significant importance. The further dynamic development of dietary systems using AI technology may lead to the creation of a global network that will be able to both actively support and monitor the personalized supply of nutrients [79]. In this case, consideration should be given to geographical and cultural differences in the management of food and nutrients. Perhaps the development of AI in nutrients research will enable the creation of personalized nutrition databases as a starting point for modulating daily nutrition, as enabled by Nutri-Educ based on fuzzy arithmetic [51].

On the basis of this review, it is worthwhile to consider the possibility of creating AI systems to coordinate both biomedical and clinical nutrients research with nutritional epidemiology. Perhaps the gut microbiota function may be an important mediator of this kind of advanced coordination. Therefore, research on the importance of the intestinal flora is of fundamental importance in the field of nutrients research. A significant challenge for the near future is the use of AI technology in the creation of gut microbiota biobanks for the purpose of scientific research [80].

Despite the fact that AI technologies are dynamically developing, the problem in nutrients research is not currently obtaining more and more advanced algorithms, but the application of those that have already been developed and are standardly used in other fields of knowledge, and even in other areas of biomedicine. An important challenge for nutrients research is also their integration with research on the use of medical robotics. Perhaps the development and application of AI in nutrients research requires modification of both mentality and professional competences, as is already postulated in relation to the food industry [81].

## Figures and Tables

**Table 1:** The characteristics of the included studies on biomedical nutrients research.

**Biomedical Nutrients Research**	**Topic**	**Number of Studies** **[Ref]**	**Nutrients**	**Domains**	**Algorithms**	**Years**
	Food composition	6 [23,24,25,26,27,28]	Proteins, Minerals (K, Ca, Mg), Trace elements	ANN, ML	SVM, LS-SVM, SVR, GA-RBFN, PLS, GA-PLS, KohNN, LASSO, CLAs	1996, 2013, 2016, 2017, 2019
	Production of nutrients	3 [29,30,31]	Retinol, Benzoquinones, Phycobiliproteins	ANN, FLM	LM, GA, ANN-GAR, FFD, GA-Fuzzy	2017, 2020
	Influence of nutrients on phys./path. functions	8 [32,33,34,35,36,37,38,39]	Proteins, Vitamins (A,B,C,D,K)	ANN, FLM, ML	SVM, BN, NB, RF, CLAs	2013, 2014, 2016, 2018, 2019
	Gut microbiota	3 [40,41,42]	Nutrients from food	ML, NV	SVM, kNN, RF, CLAs	2015, 2017, 2019
Total		20				1996–2020

Note: Domains: ANN = artificial neural network, ML = machine learning, FLM = fuzzy logic methodology, NV = network visualization; learning algorithms: kNN = k-nearest neighbor, KohNN = Kohonen neural network, LM = Levenberg–Marquardt algorithm, GA = genetic algorithm, ANN-GAR = Garson’s algorithm, GA-Fuzzy = fuzzy genetic algorithm, FFD = fractional factorial design, LASSO = least absolute shrinkage and selection operator, GA-PLS = genetic algorithm-partial least squares, PLS = partial least squares regression, GA-RBFN = genetic algorithm-radial basis function network, LS-SVM = least squares support vector machine, SVM = support vector machine, SVR = support vector regression, BN = Bayes net, NB = naive Bayes, RF = random forest, CLAs = clustering algorithms.

**Table 2:** The characteristics of the included studies on clinical nutrients research.

**Clinical Nutrients Research**	**Topic**	**Number of Studies** **[Ref]**	**Nutrients**	**Domains**	**Algorithms**	**Years**
	Clinical nutrients intake	11 [43,44,45,46,47,48,49,50,51,52,53]	Carbohydrate, Lactose, Protein, Minerals	ML, DL FLM	LASSO, FFNN, SVM, kNN,	2003, 2008, 2015, 2017–2019
	Diseases risks to food and nutrients patterns	4 [54,55,56,57]	Carbohydrate, Triglyceride, Micronutrients (folate, B12)	ANN, ML	kNN, DTA LR, RF	2016, 2018, 2020
	Disease and trace elements levels	4 [58,59,60,61]	Trace elements (lithium, zinc, chromium, copper, iron, manganese)	ML	SVM, DTA, RF, NB	2009, 2012, 2014, 2017
	Supplementations	3 [62,63,64]	Vitamins (A, C, D) Curcumin, Glycyrrhizic acid	ML	CLAs	2020
Total		22				2003–2020

Note: Domains/methods: ANN = artificial neural network, ML = machine learning, DL = deep learning, FLM = fuzzy logic methodology; learning algorithms: kNN = k-nearest neighbor, LASSO = least absolute shrinkage and selection operator, FFNN = feed forward neural network, LR = linear regression, RF = random forest, DTA = decision tree algorithm, SVM = support vector machines, NB = naive Bayes, CLAs = clustering algorithms.

**Table 3:** The characteristics of the included studies on nutritional epidemiology.

**Nutritional Epidemiology**	**Topic**	**Number of Studies** **[Ref]**	**Nutrients**	**Domains**	**Algorithms**	**Years**
	Dietary assessment	7 [65,66,67,68,69,70,71]	Macronutrients	ML, DL FLM	ICP, CLAs	2008, 2011, 2018–2020
	Physical monitoring systems	3 [72,73,74]	Macronutrients	IoT, ML, DL FLM	kNN, SVM, BDLN	2019–2020
	Environmental trace elements monitoring systems	3 [75,76,77]	Trace elements	ANN, ML	PNN, KohNN, PLS	2009, 2017, 2020
Total		13				2008–2020

Note: Domains/methods: ANN = artificial neural network, ML = machine learning, DL = deep learning, FLM = fuzzy logic methodology, IoT = Internet of Things; learning algorithms: ICP = iterative closest point algorithm, CLAs = clustering algorithms, kNN = k-nearest neighbor, SVM = support vector machine, BDLN = Bayesian deep learning network, PNN = probabilistic neural network, KohNN = Kohonen neural network, PLS = partial least squares regression.
